# Pembrolizumab plus chemotherapy in Japanese patients with triple‐negative breast cancer: Results from KEYNOTE‐355

**DOI:** 10.1002/cam4.5757

**Published:** 2023-03-14

**Authors:** Masaya Hattori, Norikazu Masuda, Toshimi Takano, Koichiro Tsugawa, Kenichi Inoue, Koji Matsumoto, Takashi Ishikawa, Mitsuya Itoh, Hiroyuki Yasojima, Yuko Tanabe, Keiko Yamamoto, Masato Suzuki, Wilbur Pan, Javier Cortes, Hiroji Iwata

**Affiliations:** ^1^ Aichi Cancer Center Hospital Nagoya Japan; ^2^ Nagoya University Graduate School of Medicine Nagoya Japan; ^3^ National Hospital Organization Osaka National Hospital Osaka Japan; ^4^ The Cancer Institute Hospital of JFCR Tokyo Japan; ^5^ Toranomon Hospital Tokyo Japan; ^6^ St. Marianna University Hospital Kawasaki Japan; ^7^ Saitama Cancer Center Saitama Japan; ^8^ Hyogo Cancer Center Hyogo Japan; ^9^ Tokyo Medical University Hospital Tokyo Japan; ^10^ Hiroshima City Hiroshima Citizens Hospital Hiroshima Japan; ^11^ MSD K.K. Tokyo Japan; ^12^ Merck & Co., Inc. Rahway New Jersey USA; ^13^ International Breast Cancer Center (IBCC), Pangaea Oncology, Quironsalud Group, Madrid and Barcelona, Spain and Faculty of Biomedical and Health Sciences, Department of Medicine Universidad Europea de Madrid Madrid Spain

**Keywords:** breast cancer, chemotherapy, clinical cancer research, clinical trials

## Abstract

Pembrolizumab plus chemotherapy improved progression‐free survival (PFS) and overall survival (OS) compared with placebo plus chemotherapy in patients with previously untreated locally recurrent inoperable or metastatic triple‐negative breast cancer with tumor programmed cell death ligand 1 (PD‐L1) combined positive score (CPS) ≥10 in the global, phase 3, randomized controlled trial KEYNOTE‐355. We report results for patients enrolled in Japan. Patients were randomized 2:1 to pembrolizumab 200 mg or placebo Q3W for 35 cycles plus chemotherapy (nab‐paclitaxel, paclitaxel, or gemcitabine–carboplatin). Primary endpoints were PFS per RECIST version 1.1 by blinded independent central review and OS in patients with PD‐L1 CPS ≥10, PD‐L1 CPS ≥1, and the intention‐to‐treat (ITT) population. No alpha was assigned to this exploratory analysis. Eighty‐seven patients were randomized in Japan (pembrolizumab plus chemotherapy, *n* = 61; placebo plus chemotherapy, *n* = 26), 66 (76%) had PD‐L1 CPS ≥1, and 28 (32%) had PD‐L1 CPS ≥10. Median time from randomization to data cutoff (June 15, 2021) was 44.7 (range, 37.2–52.9) months in the ITT population. Hazard ratios (HRs; 95% CI) for OS were 0.36 (0.14–0.89), 0.52 (0.30–0.91), and 0.46 (0.28–0.77) in the PD‐L1 CPS ≥10, PD‐L1 CPS ≥1, and ITT populations, respectively. HRs (95% CI) for PFS were 0.52 (0.20–1.34), 0.61 (0.35–1.06), and 0.64 (0.39–1.05). Grade 3 or 4 treatment‐related adverse events occurred in 85% of patients in each group (no grade 5 events). Consistent with the global population, pembrolizumab plus chemotherapy tended to show improvements in OS and PFS with manageable toxicity versus placebo plus chemotherapy in Japanese patients and supports this combination in this setting.

## Graphical abstract

Overall survival was prolonged with pembrolizumab plus chemotherapy compared with placebo plus chemotherapy among patients with previously untreated locally recurrent inoperable or metastatic triple‐negative breast cancer enrolled in the global, phase 3 randomized controlled trial KEYNOTE‐355. In this analysis of outcomes in patients in KEYNOTE‐355 who were enrolled in Japan, pembrolizumab plus chemotherapy improved overall survival versus placebo plus chemotherapy in patients with previously untreated locally recurrent inoperable or metastatic triple‐negative breast cancer with manageable toxicity.

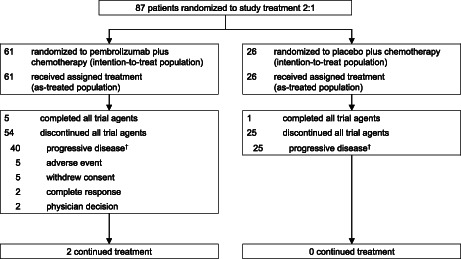

## 1 INTRODUCTION

Breast cancer was the leading cause of cancer among women in 2020, both globally and in Japan.^1^, ^2^ Breast cancer is associated with high mortality and was the fifth leading cause of cancer‐related death among women in Japan in 2019.^2^ However, Asian patients are sometimes underrepresented in global, phase 3 trials. Evidence has suggested that differences in patient characteristics and tumor pathophysiologic characteristics (such as luminal A to luminal B ratio, frequency of TP53 mutations, and tumor‐infiltrating lymphocyte gene signatures) between Asian and nonAsian patients may affect the observed response to treatment in clinical trials.^3^ Additionally, safety findings from certain trials of targeted therapies in patients with breast cancer have reported higher frequencies of adverse events (such as neutropenia and thrombocytopenia) in Asian patients than in non–Asian patients.^3^ Given these considerations, it is important to assess response to treatments specifically in Japanese patients.

Evidence has suggested that Japanese patients may have better survival outcomes following treatment than American patients with breast cancer.^4^ Such differences in treatment outcomes may be due to biological factors, treatment patterns, lifestyle, and differences in the gut microbiome (potentially due to differences in diet).^4^, ^5^, ^6^ Triple‐negative breast cancer (TNBC), defined as breast cancer that is estrogen receptor‐, progesterone receptor‐, and human epidermal growth factor receptor 2‐negative, accounts for 10%–15% of all breast cancers in Japan and is associated with a poor prognosis.^4^, ^7^, ^8^ Cytotoxic chemotherapy has historically been the mainstay standard of care for metastatic TNBC.^9^, ^10^, ^11^

Pembrolizumab, a humanized anti‐programmed cell death protein 1 (PD‐1) antibody, has demonstrated efficacy in patients with TNBC.^12^, ^13^, ^14^, ^15^ In patients with early‐stage TNBC, pembrolizumab in combination with standard‐of‐care chemotherapy demonstrated statistically significant and clinically meaningful improvement in pathological complete response and event‐free survival (primary endpoints) in the phase 3 KEYNOTE‐522 study.^14^, ^15^ Among patients with programmed cell death ligand 1 (PD‐L1)‐positive metastatic TNBC, pembrolizumab plus chemotherapy improved progression‐free survival (PFS) and overall survival (OS) outcomes compared with placebo plus chemotherapy.^12^, ^13^ In the global phase 3 KEYNOTE‐355 (NCT02819518) study of pembrolizumab plus chemotherapy versus placebo plus chemotherapy in patients with previously untreated locally recurrent inoperable or metastatic TNBC, pembrolizumab plus chemotherapy significantly improved the study's primary endpoints of PFS and OS versus placebo plus chemotherapy in patients with metastatic TNBC whose tumors expressed PD‐L1 (combined positive score [CPS] ≥10).^12^, ^13^

Among the 847 patients enrolled in KEYNOTE‐355 worldwide, 87 patients were from Japan. Given the potential for differences in response to treatment among Japanese patients, it is important to assess outcomes in this setting. Here, we report results for the subset of patients in KEYNOTE‐355 enrolled in Japan.

## 2 MATERIALS AND METHODS

### 2.1 Patient eligibility

Eligibility criteria for KEYNOTE‐355 have been previously published.^12^, ^13^ Briefly, eligible patients were ≥18 years of age, with centrally confirmed TNBC (as defined by American Society of Clinical Oncology–College of American Pathologists guidelines)^16^, ^17^ that was locally recurrent inoperable or metastatic, previously untreated with chemotherapy, and could not be treated with curative intent. Patients had completed treatment for stage I–III breast cancer (if indicated), with ≥6 months having elapsed between completion of treatment with curative intent (i.e., date of primary breast tumor surgery or date of last adjuvant chemotherapy administration including capecitabine, whichever occurred last) and first documented local or distant disease recurrence; measurable disease per Response Evaluation Criteria in Solid Tumors (RECIST) version 1.1 as assessed by the investigator; provided a newly obtained tumor sample from a locally recurrent inoperable or metastatic site for central determination of TNBC status and PD‐L1 expression; an Eastern Cooperative Oncology Group performance status (ECOG PS) of 0 or 1; and adequate organ function. Exclusion criteria included an active autoimmune disease that had required systemic treatment in the past 2 years, diagnosis of immunodeficiency or receipt of systemic steroid therapy or any other immunosuppressive therapy within 7 days before randomization, active central nervous system metastases and/or carcinomatous meningitis, and receipt of prior therapy with an anti‐PD(L)‐1 agent, anti‐PD‐L2 agent, or an agent directed to another coinhibitory T‐cell receptor.

The trial protocol was approved by an institutional review board or independent ethics committee at each site, and the trial was conducted in accordance with Good Clinical Practice guidelines and the Declaration of Helsinki. Written informed consent was obtained from all patients.

### 2.2 Study design

As previously described,^12^, ^13^ KEYNOTE‐355 (ClinicalTrials.gov, NCT02819518) was a randomized, placebo‐controlled, double‐blind, global, phase 3 study of pembrolizumab plus chemotherapy versus placebo plus chemotherapy in patients with locally recurrent inoperable or metastatic TNBC that was previously untreated with chemotherapy and could not be treated with curative intent.

Eligible patients were randomized 2:1 in a double‐blind manner to receive pembrolizumab plus chemotherapy or placebo plus chemotherapy. Pembrolizumab 200 mg IV or saline placebo was administered every 3 weeks for up to 35 cycles (approximately 2 years). Chemotherapy comprised the investigator's choice of nab‐paclitaxel 100 mg/m^2^ IV on days 1, 8, and 15 every 28 days; paclitaxel 90 mg/m^2^ IV on days 1, 8, and 15 every 28 days; or gemcitabine 1000 mg/m^2^ and carboplatin AUC 2 on days 1 and 8 every 21 days. Randomization was stratified by investigator's choice of chemotherapy (taxane vs. gemcitabine–carboplatin), tumor PD‐L1 status (CPS <1 vs. CPS ≥1), and prior treatment with the same class of chemotherapy in the neoadjuvant or adjuvant setting (yes vs. no). Study treatment continued until completion of 35 cycles (approximately 2 years) of pembrolizumab or placebo (treatment with chemotherapy could have been continued at the investigator's discretion), disease progression, unacceptable toxicity, intercurrent illness that prevented further treatment administration, investigator decision, or patient withdrawal of consent. Crossover between treatment arms was not permitted.

### 2.3 Assessments

Tumor imaging was performed at baseline, at weeks 8, 16, and 24 from randomization, then every 9 weeks for the first year and every 12 weeks thereafter. Responses were assessed by RECIST version 1.1 by blinded independent central review. Survival was assessed every 12 weeks until withdrawal of consent or end of study. Adverse events (AEs) were monitored from the time of randomization through 30 days following cessation of treatment (90 days for serious AEs) and were graded according to the National Cancer Institute Common Terminology Criteria for Adverse Events version 4.0. Baseline tumor PD‐L1 expression was assessed using PD‐L1 IHC 22C3 pharmDx (Agilent Technologies) at a central laboratory and reported as CPS, defined as the number of PD‐L1‐positive tumor cells, lymphocytes, and macrophages divided by the total number of tumor cells multiplied by 100.

### 2.4 Endpoints

The dual primary endpoints were PFS per RECIST version 1.1 as assessed by blinded independent central review and OS in patients in the intention‐to‐treat population, patients with PD‐L1 CPS ≥10 tumors, and patients with PD‐L1 CPS ≥1 tumors. The primary endpoints were amended to include PFS and OS in patients with PD‐L1 CPS ≥10 tumors following the completion of enrollment and the first interim analysis based on data from other studies that reported increased clinical benefit with PD‐L1 enrichment.^12^, ^18^, ^19^, ^20^, ^21^ Key secondary endpoints were safety in all treated patients and objective response rate (ORR) assessed by blinded independent central review in the intention‐to‐treat population, patients with PD‐L1 CPS ≥10 tumors, and patients with PD‐L1 CPS ≥1 tumors. Other secondary endpoints included duration of response and disease control rate per RECIST version 1.1 by blinded independent central review, each in the intention‐to‐treat population, in patients with PD‐L1 CPS ≥10 tumors, and in patients with PD‐L1 CPS ≥1 tumors. Analysis of outcomes among patients enrolled in Japan was exploratory.

### 2.5 Statistical analysis

Statistical considerations for the global KEYNOTE‐355 study have been previously described.^12^, ^13^ This study was not designed to test hypotheses in the Japanese subset and therefore lacks power for inferential purposes in this population. No alpha was assigned to this analysis. Efficacy was assessed in patients with PD‐L1 CPS ≥10 tumors, patients with PD‐L1 CPS ≥1 tumors, and the intention‐to‐treat population (i.e., all randomized patients). Safety was assessed in all treated patients (i.e., all randomized patients who received ≥1 dose of study treatment), and patients were included in the treatment group corresponding to the study treatment received. PFS and OS were estimated by the Kaplan–Meier method, and treatment differences were assessed by the stratified log‐rank test. HRs and corresponding 95% CIs were assessed using a Cox proportional hazards model with the Efron method of tie handling to assess the magnitude of treatment differences. Differences in ORR were assessed using the stratified Miettinen and Nurminen method.

## 3 RESULTS

### 3.1 Patients

Among 847 patients enrolled in the global study,^12^, ^13^ 87 were enrolled in Japan (pembrolizumab plus chemotherapy, *n* = 61; placebo plus chemotherapy, *n* = 26) between January 18, 2017, and May 10, 2018. In the intention‐to‐treat population (*n* = 87), 28 patients (32%) had PD‐L1 CPS ≥10 tumors and 66 (76%) had PD‐L1 CPS ≥1 tumors. Demographics and baseline disease characteristics are shown in Table 1. The majority of patients in either treatment arm had an ECOG PS of 0 and were postmenopausal.

**TABLE 1:** Demographics and baseline disease characteristics in the intention‐to‐treat population.

	Intention‐to‐treat population	
	Pembrolizumab plus chemotherapy	Placebo plus chemotherapy
	(*n* = 61)	(*n* = 26)
Age, median (range), years	54 (29–76)	51 (25–74)
<65 years of age	47 (77)	19 (73)
ECOG PS 0	52 (85)	21 (81)
ECOG PS 1	9 (15)	5 (19)
PD‐L1 CPS		
<1	17 (28)	4 (15)
≥1	44 (72)	22 (85)
≥10	19 (31)	9 (35)
Menopausal status		
Premenopausal	22 (36)	7 (27)
Postmenopausal	39 (64)	19 (73)
Disease‐free interval		
De novo metastasis	19 (31)	8 (31)
<12 month	12 (20)	3 (12)
≥12 month	30 (49)	15 (58)
Disease status		
Metastatic, de novo	19 (31)	8 (31)
Metastatic, recurrence	39 (64)	17 (65)
Locally recurrent inoperable	3 (5)	1 (4)
Chemotherapy on‐study		
Nab‐paclitaxel	5 (8)	5 (19)
Paclitaxel	7 (11)	3 (12)
Gemcitabine–carboplatin	49 (80)	18 (69)
Prior same‐class chemotherapy (IVRS)		
Yes	5 (8)	5 (19)
No	56 (92)	21 (81)

*Note*: Values are presented as n (%) unless stated otherwise.

Abbreviations: CPS, combined positive score; ECOG PS, Eastern Cooperative Oncology Group performance status; IVRS, interactive voice‐response system; PD‐L1, programmed cell death ligand 1.

**FIGURE 1:**
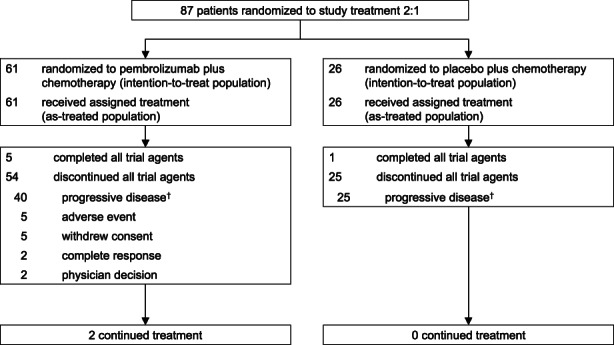
Patient disposition in the Japan subset. ^†^Includes patients with clinical progression and progressive disease.

In Japanese patients, the median time from randomization to the database cutoff date of June 15, 2021 (i.e., the same database cutoff date as the protocol‐specified final analysis for the global KEYNOTE‐355 population reported previously^13^) was 44.7 months (range, 37.2–52.9 months) in the intention‐to‐treat population. At the time of data cutoff, 54 patients (89%) in the pembrolizumab plus chemotherapy group had discontinued all study treatments, 5 patients (8%) had completed 35 cycles (approximately 2 years) of pembrolizumab, and 2 patients (3%) were continuing study treatment. The reasons for discontinuation were progressive disease (*n* = 40), AEs (*n* = 5), withdrawal of consent (*n* = 5), complete response (*n* = 2), and physician decision (*n* = 2). In the placebo plus chemotherapy group, 25 patients (96%) had discontinued all study treatments due to progressive disease, 1 patient (4%) had completed treatment, and no patients were continuing study treatment (Figure 1).

Median duration of treatment (for any component of study medication) in the pembrolizumab plus chemotherapy group and placebo plus chemotherapy group was 7.4 months (range, 0.3–44.3 months) and 7.1 months (range, 1.2 to 25.8 months), respectively. A summary of on‐study treatment exposure is provided in Table S1 and Table S2.

### 3.2 Efficacy

**FIGURE 2:**
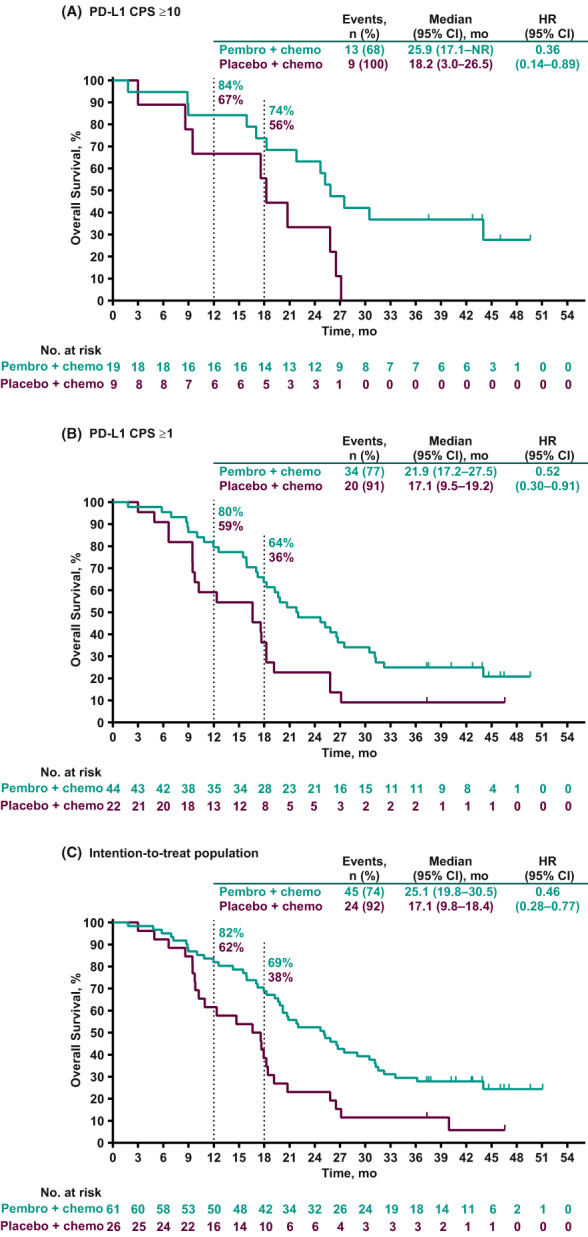
Overall survival in (A) patients with PD‐L1 CPS ≥10 tumors, (B) patients with PD‐L1 CPS ≥1 tumors, and (C) the intention‐to‐treat population. CPS, combined positive score; HR, hazard ratio; NR, not reached; PD‐L1, programmed cell death ligand 1.

Among the 87 patients in the Japan subset, 69 (79%) had died at the time of data cutoff. In the 28 patients with PD‐L1 CPS ≥10 tumors, the median OS was 25.9 months (95% CI, 17.1 months–not reached) in the pembrolizumab plus chemotherapy group and 18.2 months (95% CI, 3.0–26.5 months) in the placebo plus chemotherapy group (HR, 0.36 [95% CI, 0.14–0.89]). The 18‐month OS rates were 74% and 56%, respectively (Figure 2A). In the 66 patients with PD‐L1 CPS ≥1 tumors, the median OS was 21.9 months (95% CI, 17.2–27.5 months) versus 17.1 months (95% CI, 9.5–19.2 months) in the pembrolizumab plus chemotherapy group versus placebo plus chemotherapy group, respectively (HR, 0.52 [95% CI, 0.30–0.91]); the respective 18‐month OS rates were 64% and 36% (Figure 2B). In the intention‐to‐treat population, the median OS was 25.1 months (95% CI, 19.8–30.5 months) in the pembrolizumab plus chemotherapy group and 17.1 months (95% CI, 9.8–18.4 months) in the placebo plus chemotherapy group (HR, 0.46 [95% CI, 0.28–0.77]). The 18‐month OS rates were 69% and 38%, respectively (Figure 2C).

**FIGURE 3:**
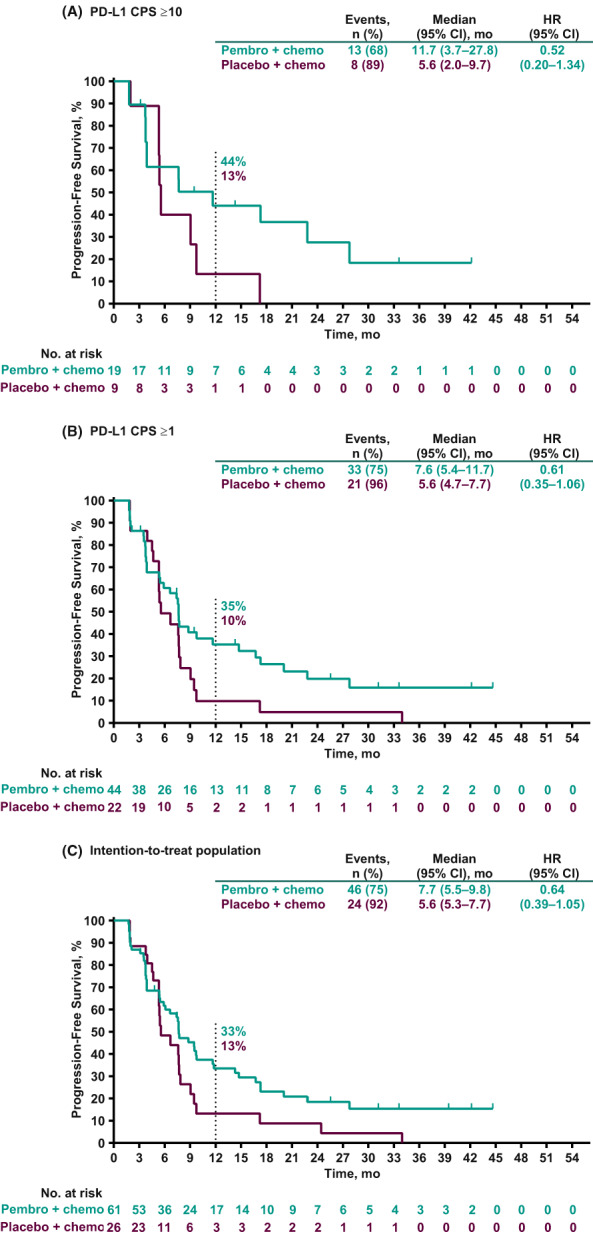
Progression‐free survival per RECIST version 1.1 by blinded independent central review in (A) patients with PD‐L1 CPS ≥10 tumors, (B) patients with PD‐L1 CPS ≥1 tumors, and (C) the intention‐to‐treat population. CPS, combined positive score; HR, hazard ratio; PD‐L1, programmed cell death ligand 1.

Among all 87 patients in the Japan subset, 70 (80%) had experienced a PFS event at the time of data cutoff. Among patients with PD‐L1 CPS ≥10 tumors, the median PFS was 11.7 months (95% CI, 3.7–27.8 months) in the pembrolizumab plus chemotherapy group and 5.6 months (95% CI, 2.0–9.7 months) in the placebo plus chemotherapy group (HR, 0.52 [95% CI, 0.20–1.34]). The 12‐month PFS rates were 44% and 13%, respectively (Figure 3A). In patients with PD‐L1 CPS ≥1 tumors, the median PFS was 7.6 months (95% CI, 5.4–11.7 months) versus 5.6 months (95% CI, 4.7–7.7 months) in the pembrolizumab plus chemotherapy group versus the placebo plus chemotherapy group, respectively (HR, 0.61 [95% CI, 0.35–1.06]), with corresponding 12‐month PFS rates of 35% and 10% (Figure 3B). In the intention‐to‐treat population, the median PFS was 7.7 months (95% CI, 5.5–9.8 months) in the pembrolizumab plus chemotherapy group and 5.6 months (95% CI, 5.3–7.7 months) in the placebo plus chemotherapy group (HR, 0.64 [95% CI, 0.39–1.05]). The respective 12‐month PFS rates were 33% and 13% (Figure 3C).

Among patients with PD‐L1 CPS ≥10 tumors, the ORR per RECIST version 1.1 by blinded independent central review was 42% (95% CI, 20–67) in the pembrolizumab plus chemotherapy group and 33% (95% CI, 7–70) in the placebo plus chemotherapy group. The median duration of response was 19.3 months (range, 6.0–40.3+ months) in the pembrolizumab plus chemotherapy group and 7.3 months (range, 3.4–15.4 months) in the placebo plus chemotherapy group. In patients with PD‐L1 CPS ≥1 tumors, the ORR was 43% (95% CI, 28–59) and 50% (95% CI, 28–72) and the median duration of response was 14.9 months (range, 3.5–42.8+ months) and 6.0 months (range, 2.3–28.5 months), respectively. In the intention‐to‐treat population, the ORR was 43% (95% CI, 30–56) in the pembrolizumab plus chemotherapy group and 46% (95% CI, 27–67) in the placebo plus chemotherapy group. The median duration of response was 12.6 months (range, 3.0+ to 42.8+ months) and 6.0 months (range, 2.3–28.5 months), respectively. The disease control rates were similar between the treatment groups across all 3 populations (Table 2).

**TABLE 2:** Summary of confirmed ORR per RECIST Version 1.1 by blinded independent central review.

	PD‐L1 CPS ≥10		PD‐L1 CPS ≥1		Intention‐to‐treat population	
	Pembrolizumab plus chemotherapy	Placebo plus chemotherapy	Pembrolizumab plus chemotherapy	Placebo plus chemotherapy	Pembrolizumab plus chemotherapy	Placebo plus chemotherapy
	(*n* = 19)	(*n* = 9)	(*n* = 44)	(*n* = 22)	(*n* = 61)	(*n* = 26)
ORR, % (95% CI)	42 (20–67)	33 (7–70)	43 (28–59)	50 (28–72)	43 (30–56)	46 (27–67)
Disease control rate, % (95% CI)	58 (33–80)	56 (21–86)	64 (48–78)	64 (41–83)	64 (51–76)	58 (37–77)
Best overall response						
Complete response	3 (16)	1 (11)	6 (14)	1 (5)	7 (11)	1 (4)
Partial response	5 (26)	2 (22)	13 (30)	10 (45)	19 (31)	11 (42)
Stable disease	9 (47)	5 (56)	19 (43)	8 (36)	27 (44)	11 (42)
Progressive disease	1 (5)	1 (11)	5 (11)	3 (14)	7 (11)	3 (12)
Not evaluable^a^	0	0	0	0	0	0
No assessment^b^	1 (5)	0	1 (2)	0	1 (2)	0
Median duration of response (range),^c^ months	19.3 (6.0–40.3^d^)	7.3 (3.4–15.4)	14.9 (3.5–42.8^d^)	6.0 (2.3–28.5)	12.6 (3.0 ± 42.8^d^)	6.0 (2.3–28.5)
Patients with extended response duration, %^c^						
≥6 months	100	67	78	55	79	58
≥12 months	73	33	55	18	52	25

*Note*: Values are presented as n (%) unless stated otherwise.

Abbreviations: CPS, combined positive score; ORR, overall response rate; PD‐L1, programmed cell death ligand 1; RECIST, Response Evaluation Criteria in Solid Tumors.

^a^ Not evaluable includes subjects with insufficient data for assessment of response per RECIST version 1.1.

^b^ No assessment includes subjects without postbaseline assessment on the data cutoff date.

^c^ From product‐limit (Kaplan–Meier) method for censored data.

^d^ No progressive disease by the time of last disease assessment.

### 3.3 Safety

Treatment‐related AEs occurred in all 61 patients (100%) and 26 patients (100%) in the pembrolizumab plus chemotherapy and placebo plus chemotherapy groups, respectively. The most common treatment‐related AEs in both treatment groups were decreased white blood cell count (pembrolizumab plus chemotherapy, 75%; placebo plus chemotherapy, 85%), decreased neutrophil count (72% and 81%), anemia (66% and 62%), and nausea (52% and 62%). Grade 3 or 4 treatment‐related AEs occurred in 52 patients (85%) in the pembrolizumab plus chemotherapy group and 22 (85%) in the placebo plus chemotherapy group. The most frequently occurring grade 3 or 4 treatment‐related AEs in the pembrolizumab plus chemotherapy group versus placebo plus chemotherapy group were decreased neutrophil count (59% vs. 62%), decreased white blood cell count (49% vs. 58%), and anemia (26% vs. 23%). Nineteen patients (31%) in the pembrolizumab plus chemotherapy group and four patients (15%) in the placebo plus chemotherapy group discontinued any treatment due to a treatment‐related AE. No patients in either group died due to a treatment‐related AE.

Immune‐mediated AEs and infusion reactions of any grade occurred in 20 patients (33%) in the pembrolizumab plus chemotherapy group and four patients (15%) in the placebo plus chemotherapy group. The most frequently occurring immune‐mediated AEs (incidence ≥5%) in the pembrolizumab plus chemotherapy group were hypothyroidism (*n* = 7, 11%) and adrenal insufficiency (*n* = 5, 8%). In the placebo plus chemotherapy group, immune‐mediated AEs were hyperthyroidism and vasculitis (*n* = 1 each, 3.8%). Ten patients (16%) in the pembrolizumab plus chemotherapy group and two patients (8%) in the placebo plus chemotherapy group had infusion reactions. Grade 3 or 4 immune‐mediated AEs and infusion reactions occurred in four patients (7%) in the pembrolizumab plus chemotherapy group (grade 3 adrenal insufficiency, *n* = 2; grade 4 infusion reaction, *n* = 1; grade 3 severe skin reaction, *n* = 1); there were no grade 3 or 4 events in the placebo plus chemotherapy group. Five patients (8%) in the pembrolizumab plus chemotherapy group and two patients (8%) in the placebo plus chemotherapy group discontinued any treatment due to an immune‐mediated AE. No patients in either group died due to an immune‐mediated AE or infusion reaction (Table 3).

**TABLE 3:** Summary of AEs in all treated patients.

	Pembrolizumab plus chemotherapy	Placebo plus chemotherapy
	*n* = 61	*n* = 26
Any AE	61 (100)	26 (100)
Treatment‐related AE		
Any grade	61 (100)	26 (100)
Grade 3–5^a^	52 (85)	22 (85)
Led to death	0	0
Led to discontinuation	19 (31)	4 (15)
Any AE leading to dose modification		
Pembrolizumab or placebo	40 (66)	17 (65)
Nab‐paclitaxel	5 (8)	3 (12)
Paclitaxel	6 (10)	2 (8)
Gemcitabine	46 (75)	17 (65)
Carboplatin	46 (75)	17 (65)

	Any grade	Grade 3/4	Any grade	Grade 3/4
Treatment‐related AEs with incidence ≥20%				
Decreased white blood cell count	46 (75)	30 (49)	22 (85)	15 (58)
Decreased neutrophil count	44 (72)	36 (59)	21 (81)	16 (62)
Anemia	40 (66)	16 (26)	16 (62)	6 (23)
Nausea	32 (52)	1 (2)	16 (62)	1 (4)
Alopecia	25 (41)	0	10 (38)	0
Constipation	24 (39)	1 (2)	10 (38)	0
Malaise	22 (36)	2 (3)	12 (46)	0
Dysgeusia	20 (33)	0	1 (4)	0
Decreased platelet count	20 (33)	6 (10)	8 (31)	4 (15)
Decreased appetite	19 (31)	1 (2)	4 (15)	1 (4)
Stomatitis	18 (30)	0	4 (15)	0
Peripheral sensory neuropathy	16 (26)	1 (2)	6 (23)	0
Fatigue	15 (25)	1 (2)	6 (23)	1 (4)
Rash	13 (21)	0	1 (4)	0
Immune‐mediated AEs and infusion reactions^a^				
Any	20 (33)	4 (7)	4 (15)	0
Infusion reactions	10 (16)	1 (2)	2 (8)	0
Hypothyroidism	7 (11)	0	0	0
Adrenal insufficiency	5 (8)	2 (3)	0	0
Hyperthyroidism	3 (5)	0	1 (4)	0
Pneumonitis	1 (2)	0	0	0
Severe skin reactions	1 (2)	1 (2)	0	0
Thyroiditis	1 (2)	0	0	0
Vasculitis	1 (2)	0	1 (4)	0

*Note*: All values are presented as *n* (%).

Abbreviation: AE, adverse event.

^a^ There were no grade 5 treatment‐related AEs, immune‐mediated AEs, or infusion reactions in either treatment group.

## 4 DISCUSSION

In this subset analysis of patients in the phase 3 KEYNOTE‐355 study enrolled in Japan, pembrolizumab plus chemotherapy improved outcomes versus placebo plus chemotherapy in patients with previously untreated locally recurrent inoperable or metastatic TNBC. Baseline characteristics were generally consistent among Japanese patients and those enrolled in the global population.^12^, ^13^ A slightly higher proportion of patients enrolled in Japan had an ECOG PS of 0 (pembrolizumab plus chemotherapy, 85%; placebo plus chemotherapy, 81%) compared with the global population (59% and 62%), and slightly higher proportions had received gemcitabine–carboplatin as chemotherapy on‐study (pembrolizumab plus chemotherapy, 80%; placebo plus chemotherapy, 69%) compared with the global study (55% and 55%). A broad body of evidence supports the efficacy of gemcitabine–platinum combinations in patients with metastatic TNBC,^12^, ^22^, ^23^, ^24^ which may explain, at least in part, why Japan investigators were interested in the gemcitabine–carboplatin combination and used it more at a higher rate than in the study overall, despite it not being a standard‐of‐care therapy in Japan.^9^ Treatment with pembrolizumab plus chemotherapy improved both OS and PFS, and the 18‐month OS rates and 12‐month PFS rates were higher in the pembrolizumab plus chemotherapy group than in the placebo plus chemotherapy group. The ORR and disease control rates were generally similar between the treatment groups; however, the duration of response was more than doubled among responders in the pembrolizumab plus chemotherapy group compared with the placebo plus chemotherapy group. Overall, toxicity was manageable. Findings from this analysis are generally consistent with those from the global KEYNOTE‐355 population and support the use of pembrolizumab plus chemotherapy as a new standard‐of‐care treatment regimen for patients with previously untreated locally recurrent inoperable or metastatic TNBC in Japan.^12^, ^13^

The magnitude of treatment benefit with pembrolizumab plus chemotherapy versus placebo plus chemotherapy appeared greater in Japanese patients than in the global study across each of the PD‐L1 CPS populations (i.e., PD‐L1 CPS ≥10, PD‐L1 CPS ≥1, and the intention‐to‐treat population).^12^, ^13^ Notably, the 18‐month OS rates were higher in patients in the pembrolizumab plus chemotherapy group versus the placebo plus chemotherapy group in the Japanese subset; the 18‐month OS rates were approximately doubled in each of the PD‐L1 CPS populations. However, given the wide 95% CIs for OS and PFS in the Japan subset due to smaller patient numbers than in the global study, particularly among Japanese patients with PD‐L1 CPS ≥10 (*n* = 19 and *n* = 9, respectively), we are unable to draw definitive conclusions from this data set. The reasons for the differences in OS and PFS outcomes for patients enrolled in Japan compared with the overall study population are uncertain; possible contributing factors may include the small number of patients and differences in patient baseline characteristics. The greater proportion of patients with ECOG PS of 0 in the Japanese subgroup compared with the global population (84% vs. 60%) may have contributed to the longer median OS times among the Japanese population versus the global population.

The ORRs and disease control rates were generally similar between the treatment groups in the Japan subset and the global study in each of the PD‐L1 CPS populations.^12^, ^13^ The duration of response was slightly longer in the Japan subset than in the global study, particularly in patients with PD‐L1 CPS ≥10 tumors; however, it is important to note the very small number of responders with PD‐L1 CPS ≥10 tumors in the Japan subset (pembrolizumab plus chemotherapy, *n* = 8; placebo plus chemotherapy, *n* = 3).

The toxicity profile was generally consistent with the profile reported for the global study.^12^, ^13^ In the Japan subset, a slightly higher incidence of grade 3 or 4 treatment‐related AEs was reported in both treatment groups (Japan, 85% in both treatment groups; global study, 68% in pembrolizumab plus chemotherapy group vs. 67% in placebo plus chemotherapy group), and more patients in the pembrolizumab plus chemotherapy group discontinued due to treatment‐related AEs (31%) compared with the global study (18%). The higher incidence of grade 3 or 4 treatment‐related AEs and discontinuations due to AEs in the Japan subset may be attributed to differences in clinical characteristics; in particular, a higher proportion of patients in the Japan subset had received gemcitabine–carboplatin as chemotherapy on‐study. Additionally, in the Japan subgroup, 8% of patients in the pembrolizumab plus chemotherapy group experienced adrenal insufficiency (grade 3 or 4, 3%) compared with none in the placebo plus chemotherapy. Although immune‐mediated AEs and infusion reactions occurred more frequently in the pembrolizumab plus chemotherapy group than in the placebo plus chemotherapy group few of these were grade 3 or 4 and, notably, a similar proportion of patients in either treatment arm discontinued any treatment due to an immune‐mediated AE or infusion reaction.

In a subgroup analysis of Japanese patients (*n* = 65) enrolled in the phase 3 IMpassion130 study that evaluated first‐line atezolizumab (an anti‐PD‐L1 inhibitor) in combination with nab‐paclitaxel in patients with locally advanced or metastatic TNBC, the median PFS was 7.4 months (95% CI, 5.4–10.8 months) in the atezolizumab plus nab‐paclitaxel group versus 4.6 months (95% CI, 3.7–7.2 months) in the placebo plus nab‐paclitaxel group (HR, 0.47 [95% CI, 0.25–0.90]), and the median OS was not estimable (NE) versus 16.8 months (95% CI, 13.3 months–NE), respectively (HR, 0.44 [95% CI, 0.16–1.24]).^25^ As with results from KEYNOTE‐355, these outcomes provide evidence for activity of anti‐PD‐(L)1 agents in Japanese patients with advanced TNBC.

A limitation of this study is that the Japanese subset comprises a portion of patients enrolled globally in KEYNOTE‐355 (87 of 847 patients) and no alpha was allocated to this analysis. Secondly, given the small number of patients in this analysis, we were not able to perform any meaningful subset analyses, including assessments of clinical benefit with different routinely used chemotherapy regimens, assessment of the influence of differences in clinical characteristics on outcomes, or differences in treatment patterns. In the current study, the allocation of patients to nab‐paclitaxel, paclitaxel, or gemcitabine–carboplatin was by investigator's choice. At the time of writing, there is no unequivocal evidence for whether either of these chemotherapy regimens provides more clinical benefit than the other in this setting.^11^ The investigators' choice of chemotherapy regimen may have been driven, at least in part, by the regulatory and clinical guideline status of these drugs in Japan; in particular, it is important to note that platinum chemotherapeutics are not currently included as standard of care for metastatic TNBC in Japan.^9^ Nonetheless, it is apparent that approval of the combination of pembrolizumab plus chemotherapy (based on results from KEYNOTE‐355) has added to the treatment options for patients with PD‐L1‐positive TNBC in Japan.^26^

In Japanese patients enrolled in KEYNOTE‐355, pembrolizumab plus chemotherapy tended to show improvements in OS and PFS with manageable toxicity versus placebo plus chemotherapy, consistent with the global population. These findings provide support for the use of pembrolizumab plus chemotherapy in Japanese patients with previously untreated locally recurrent inoperable or metastatic TNBC.

## AUTHOR CONTRIBUTIONS

**Masaya Hattori:** Investigation (equal); validation (equal); writing – review and editing (equal). **Norikazu Masuda:** Investigation (equal); validation (equal); writing – review and editing (equal). **Toshimi Takano:** Investigation (equal); validation (equal); writing – review and editing (equal). **Koichiro Tsugawa:** Investigation (equal); validation (equal); writing – review and editing (equal). **Kenichi Inoue:** Investigation (equal); writing – review and editing (equal). **Koji Matsumoto:** Investigation (equal); resources (equal); validation (equal); writing – review and editing (equal). **Takashi Ishikawa:** Investigation (equal); resources (equal); writing – review and editing (equal). **Mitsuya Itoh:** Investigation (equal); resources (equal); writing – review and editing (equal). **Hiroyuki Yasojima:** Investigation (equal); writing – original draft (equal); writing – review and editing (equal). **Yuko Tanabe:** Investigation (equal); validation (equal); writing – review and editing (equal). **Keiko Yamamoto:** Investigation (equal); validation (equal); writing – review and editing (equal). **Masato Suzuki:** Formal analysis (equal); writing – review and editing (equal). **Wilbur Pan:** Formal analysis (equal); investigation (equal); validation (equal); writing – review and editing (equal). **Javier Cortes:** Conceptualization (equal); writing – review and editing (equal). **Hiroji Iwata:** Investigation (equal); validation (equal); writing – review and editing (equal).

## FUNDING INFORMATION

Funding for this research was provided by Merck Sharp & Dohme LLC, a subsidiary of Merck & Co., Inc., Rahway, NJ, USA.

## CONFLICT OF INTEREST STATEMENT

Masaya Hattori has received honoraria from Eli Lilly and Daiichi Sankyo. Norikazu Masuda has provided leadership to Japan Breast Cancer Research Group Association (JBCRG); has received honoraria from AstraZeneca, Chugai Pharma, Eisai, Lilly Japan, and Pfizer; and has received research funding (all to institution) from AstraZeneca, Chugai Pharma, Daiichi Sankyo, Eisai, Eli‐Lilly, Kyowa‐Kirin, MSD, Novartis, Pfizer, and Sanofi. Toshimi Takano has received honoraria from Daiichi‐Sankyo, Chugai, Eisai, Eli Lilly, and Celltrion and has received research funding (all to institution) from MSD, Daiichi‐Sankyo, Chugai, Eisai, and Ono. Koichiro Tsugawa has received manuscript fees from Pfizer and Eli Lilly; has received research funding from Konica Minolta, Inc; and has received scholarship endowments/research grants from Taiho Pharmaceutical Co., ltd and Chugai Pharmaceutical Co., ltd. Kenichi Inoue has received research funding (all to institution) from Astellas, AstraZeneca, Chugai Pharma, Daiichi Sankyo, Eli‐Lilly, MSD, Novartis, Ono, Pfizer, Sanofi, Takeda, and Taiho. Koji Matsumoto has received honoraria from Kyowa‐Kirin and Chugai and has received research funding from MSD, Eli Lilly, Daiichi‐Sankyo, Chugai, Eisai, and ICON‐Japan. Takashi Ishikawa has received honoraria from Daiichi Sankyo, Kyowa Kirin, Pfizer, and Chugai. Mitsuya Itoh has no conflicts of interest. Hiroyuki Yasojima has no conflicts of interest. Yuko Tanabe has received research funding from MSD. Keiko Yamamoto is an employee of MSD K.K., Tokyo, Japan. Masato Suzuki is an employee of MSD K.K., Tokyo, Japan. Wilbur Pan is an employee of Merck Sharp & Dohme LLC, a subsidiary of Merck & Co., Inc., Rahway, NJ, USA and stockholder in Merck & Co., Inc., Rahway, NJ, USA. Javier Cortes has been a consultant/advisor for Roche, Celgene, Cellestia, AstraZeneca, Seattle Genetics, Daiichi Sankyo, Erytech, Athenex, Polyphor, Lilly, Merck Sharp & Dohme, GSK, Leuko, Bioasis, Clovis Oncology, Boehringer Ingelheim, Ellipses, Hibercell, BioInvent, Gemoab, Gilead, Menarini, Zymeworks, and Reveal Genomics; has received honoraria from Roche, Novartis, Celgene, Eisai, Pfizer, Samsung Bioepis, Lilly, Merck Sharp & Dohme, and Daiichi Sankyo; has received research funding (all to institution) from Roche, Ariad pharmaceuticals, AstraZeneca, Baxalta GMBH/Servier Affaires, Bayer healthcare, Eisai, F.Hoffman‐La Roche, Guardant health, Merck Sharp & Dohme, Pfizer, Piqur Therapeutics, Puma C, and Queen Mary University of London; has stock in MedSIR, Nektar Pharmaceuticals, and Leuko (relative); and has received travel/accommodation expenses from Roche, Novartis, Eisai, Pfizer, Daiichi Sankyo, Astrazeneca, and Gilead. In addition, Javier Cortes holds the following patents: (1) Pharmaceutical Combinations of A Pi3k Inhibitor And A Microtubule Destabilizing Agent. Javier Cortés Castán, Alejandro Piris Giménez, Violeta Serra Elizalde. WO 2014/199294 A and (2) Her2 as a predictor of response to dual HER2 blockade in the absence of cytotoxic therapy. Aleix Prat, Antonio Llombart, Javier Cortés. US 2019/ 0338368 A1. Hiroji Iwata has no conflicts of interest. The study was funded by Merck Sharp & Dohme LLC, a subsidiary of Merck & Co., Inc., Rahway, NJ, USA. All authors had access to the data from the study and had final responsibility for the decision to submit for publication.

## ETHICS STATEMENT

The trial protocol was approved by an institutional review board or independent ethics committee at each site, and the trial was conducted in accordance with Good Clinical Practice guidelines and the Declaration of Helsinki.

## PATIENT CONSENT STATEMENT

Written informed consent was obtained from all patients.

## PERMISSION TO REPRODUCE MATERIAL FROM OTHER SOURCES

Not applicable.

## Supporting information


Table S1.

## Data Availability

Merck Sharp & Dohme LLC, a subsidiary of Merck & Co., Inc., Rahway, NJ, USA (MSD) is committed to providing qualified scientific researchers access to anonymized data and clinical study reports from the company's clinical trials for the purpose of conducting legitimate scientific research. MSD is also obligated to protect the rights and privacy of trial participants and, as such, has a procedure in place for evaluating and fulfilling requests for sharing company clinical trial data with qualified external scientific researchers. The MSD data sharing website (available at: http://engagezone.msd.com/ds_documentation.php) outlines the process and requirements for submitting a data request. Applications will be promptly assessed for completeness and policy compliance. Feasible requests will be reviewed by a committee of MSD subject matter experts to assess the scientific validity of the request and the qualifications of the requestors. In line with data privacy legislation, submitters of approved requests must enter into a standard data‐sharing agreement with MSD before data access is granted. Data will be made available for request after product approval in the US and EU or after product development is discontinued. There are circumstances that may prevent MSD from sharing requested data, including country or region‐specific regulations. If the request is declined, it will be communicated to the investigator. Access to genetic or exploratory biomarker data requires a detailed, hypothesis‐driven statistical analysis plan that is collaboratively developed by the requestor and MSD subject matter experts; after approval of the statistical analysis plan and execution of a data‐sharing agreement, MSD will either perform the proposed analyses and share the results with the requestor or will construct biomarker covariates and add them to a file with clinical data that is uploaded to an analysis portal so that the requestor can perform the proposed analyses.
