# Supplementary material for: Pembrolizumab plus chemotherapy in Japanese patients with triple‐negative breast cancer: Results from KEYNOTE‐355
Source: Cancer Med. 2023 Mar 14;12(9):10280–93. doi: 10.1002/cam4.5757 (PMC10225213; doi:10.1002/cam4.5757)
Supplement: Supplementary file 1 — Table S1. [file CAM4-12-10280-s001.docx]

## SUPPLEMENTAL MATERIAL

Table S1. Summary of On-Study Chemotherapy^†^

**Table S2.** Number of Treatment Administrations (All Patients as Treated)^†^

## **Table S1.** Summary of On-Study Chemotherapy^†^

|  | PD-L1 CPS ≥10 | | PD-L1 CPS ≥1 | | Intention-To-Treat Population | |
| --- | --- | --- | --- | --- | --- | --- |
|  | Pembrolizumab Plus Chemotherapy  (n = 19) | Placebo Plus Chemotherapy  (n = 9) | **Pembrolizumab Plus Chemotherapy**  (n = 44) | Placebo Plus Chemotherapy  (n = 22) | Pembrolizumab Plus Chemotherapy  (n = 61) | Placebo Plus Chemotherapy  (n = 26) |
| Gemcitabine–carboplatin | 14 (74) | 5 (56) | 35 (80) | 15 (68) | 49 (80) | 18 (69) |
| Nab-paclitaxel | 2 (11) | 3 (33) | 4 (9) | 5 (23) | 5 (8) | 5 (19) |
| Paclitaxel | 3 (16) | 1 (11) | 5 (11) | 2 (9) | 7 (11) | 3 (12) |

^†^Data are presented as n (%).

## Table S2. Number of Treatment Administrations (All Patients as Treated)^†^

| **Treatment Component** | Pembrolizumab Plus Chemotherapy  (n = 61) | Placebo Plus Chemotherapy  (n = 26) |
| --- | --- | --- |
| Pembrolizumab/placebo | 11 (1–35) | 10.5 (2–35) |
| Nab-paclitaxel | 30 (2–90) | 16 (6–37) |
| Paclitaxel | 18 (12–30) | 26 (14–33) |
| Gemcitabine | 16 (2–70) | 16 (4–51) |
| Carboplatin | 16 (2–68) | 16 (4–51) |

^†^Data are presented as median (range) number of administrations.
